# Probing the Binding Sites of Antibiotic Drugs Doxorubicin and *N*-(trifluoroacetyl) Doxorubicin with Human and Bovine Serum Albumins

**DOI:** 10.1371/journal.pone.0043814

**Published:** 2012-08-24

**Authors:** Daniel Agudelo, Philippe Bourassa, Julie Bruneau, Gervais Bérubé, Éric Asselin, Heidar-Ali Tajmir-Riahi

**Affiliations:** Département de Chimie-Biologie, Université du Québec à Trois-Rivières, Trois-Rivières (Québec), Canada; Concordia University Wisconsin, United States of America

## Abstract

We located the binding sites of doxorubicin (DOX) and *N*-(trifluoroacetyl) doxorubicin (FDOX) with bovine serum albumin (BSA) and human serum albumins (HSA) at physiological conditions, using constant protein concentration and various drug contents. FTIR, CD and fluorescence spectroscopic methods as well as molecular modeling were used to analyse drug binding sites, the binding constant and the effect of drug complexation on BSA and HSA stability and conformations. Structural analysis showed that doxorubicin and *N*-(trifluoroacetyl) doxorubicin bind strongly to BSA and HSA *via* hydrophilic and hydrophobic contacts with overall binding constants of *K*_DOX-BSA_ = 7.8 (±0.7)×10^3^ M^−1^, *K*_FDOX-BSA_ = 4.8 (±0.5)×10^3^ M^−1^ and *K*_DOX-HSA_ = 1.1 (±0.3)×10^4^ M^−1^, *K*_FDOX-HSA_ = 8.3 (±0.6)×10^3^ M^−1^. The number of bound drug molecules per protein is 1.5 (DOX-BSA), 1.3 (FDOX-BSA) 1.5 (DOX-HSA), 0.9 (FDOX-HSA) in these drug-protein complexes. Docking studies showed the participation of several amino acids in drug-protein complexation, which stabilized by H-bonding systems. The order of drug-protein binding is DOX-HSA > FDOX-HSA > DOX-BSA > FDOX>BSA. Drug complexation alters protein conformation by a major reduction of α-helix from 63% (free BSA) to 47–44% (drug-complex) and 57% (free HSA) to 51–40% (drug-complex) inducing a partial protein destabilization. Doxorubicin and its derivative can be transported by BSA and HSA in vitro.

## Introduction

**Figure 1:**
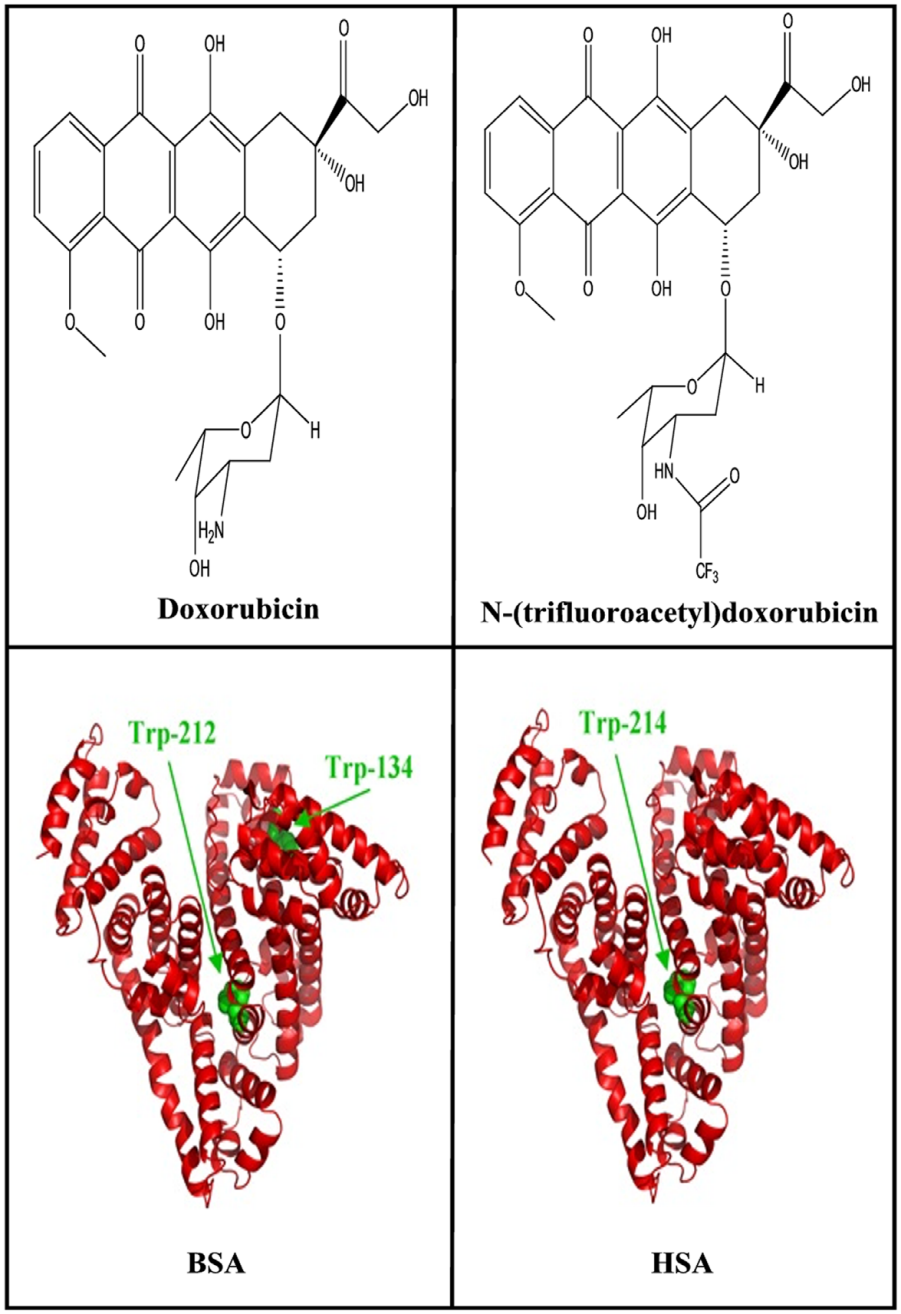
Chemical structures of doxorubicin, *N*-(trifluoroacetyl) doxorubicin and BSA and HSA with tryptophan residues in green color.

Doxorubicin (Fig. 1) is an effective chemotherapeutic agent for the treatment of breast cancer, malignant lymphomas, soft tissue sarcoma and various neoplastic diseases. The injury to non-targeted tissues often complicates cancer treatment by limiting doxorubicin dosage and diminishing the quality of patients’ life during and after doxorubicin treatment [1]. Furthermore, the use of doxorubicin has been limited by a dose-related and irreversible cardiotoxicity as well as by the emergence of drug resistance [1]. Nano-particle delivery systems of doxorubicin are a promising approach to increase its antineoplastic efficacy and lower its side-effects by site-specific drug delivery via active targeting mechanism [2], [3]. Several delivery systems have been tested for doxorubicin transport using various nanoparticles by chemical conjugation or physical encapsulation [4]–[10]. Polymer conjugates such as PEG-albumins have been recently used for distribution of doxorubicin *in vivo* [11]. Serum albumins are also used for drug delivery *in vitro* [12].

Serum albumins are the major soluble protein constituents of the circulatory system and have many physiological functions [13]. The most important property of this group of proteins is that they serve as transporters for a variety of organic and inorganic compounds including metal ions. BSA (Fig. 1) has been one of the most extensively studied of this group of proteins, particularly because of its structural homology with human serum albumin (HSA). The BSA molecule is made up of three homologous domains (I, II, III) which are divided into nine loops (L1–L9) by 17 disulfide bonds. The loops in each domain are made up of a sequence of large-small-large loops forming a triplet. Each domain in turn is the product of two subdomains (IA, IB, etc.). X-crystallographic data [14] show that the albumin structure is predominantly α-helical with the remaining polypeptide occurring in turns and extended or flexible regions between subdomains with no β-sheets. BSA (Fig. 1) has two tryptophan residues that possess intrinsic fluorescence [15]. Trp-134 in the first domain and Trp-212 in the second domain. Trp-212 is located within a hydrophobic binding pocket of the protein and Trp-134 is located on the surface of the molecule. HSA (Fig. 1) is a globular protein composed of three structurally similar domains (I, II and III), each containing two subdomains (A and B) and stabilized by 17 disulphide bridges [15], [16]. Aromatic and heterocyclic ligands were found to bind within two hydrophobic pockets in subdomains IIA and IIIA, namely site I and site II [15]–[16]. Seven binding sites for fatty acids are localized in subdomains IB, IIIA, IIIB and on the subdomain interfaces [15]. While there are marked similarities between BSA and HSA in their compositions (Fig. 1), HSA has only one tryptophan residue Trp-214, while BSA contains two tryptophan residues Trp-212 and Trp-134 that can be used as fluorophores.

Fluorescence quenching is considered as a useful method for measuring binding affinities. Fluorescence quenching is the decrease of the quantum yield of fluorescence from a fluorophore induced by a variety of molecular interactions with quencher molecule [17], [18]. Therefore, using the quenching of the intrinsic tryptophan fluorescence of BSA (Trp-212 and Trp-134) and HSA (Trp-214) as a probing tool allows us to study the interactions of doxorubicin and its derivative with serum proteins in an attempt to characterize the nature of drug-protein complexation.

**Figure 2:**
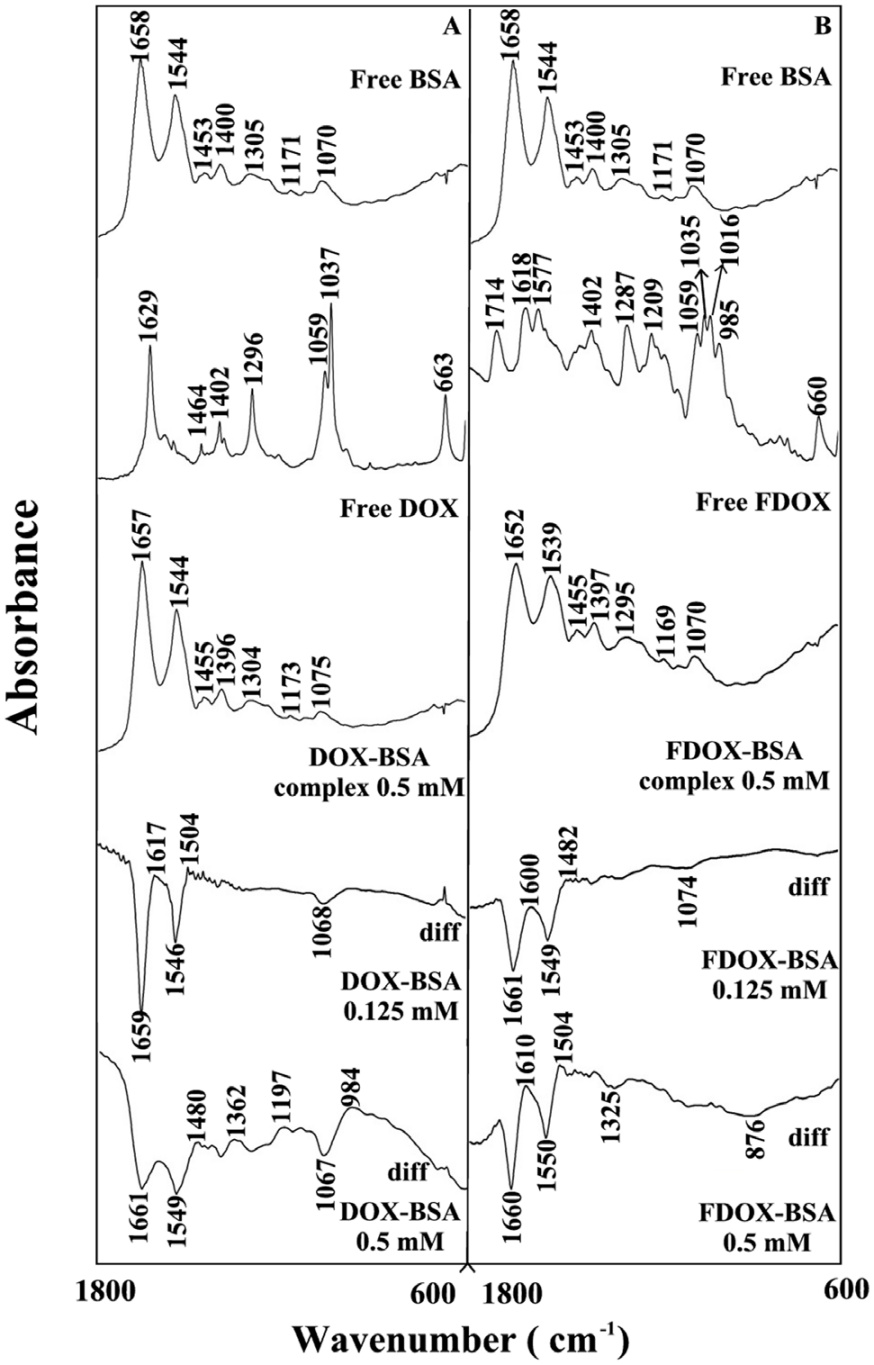
FTIR spectra in the region of 1800–600 cm^−1^ of hydrated films (pH 7.4) for free BSA (0.25 mM) and its drug complexes with difference spectra (diff.) (bottom two curves) obtained at different drug concentrations (indicated on the figure).

In this report, we present spectroscopic analysis and docking studies of the interaction of doxorubicin and *N*-(trifluoroacetyl) doxorubicin with HSA and BSA in aqueous solution at physiological conditions, using constant protein concentration and various drug contents. Structural information regarding drug binding site and the effect of drug-protein complexation on the stability and conformation of BSA and HSA is also reported here.

**Figure 3:**
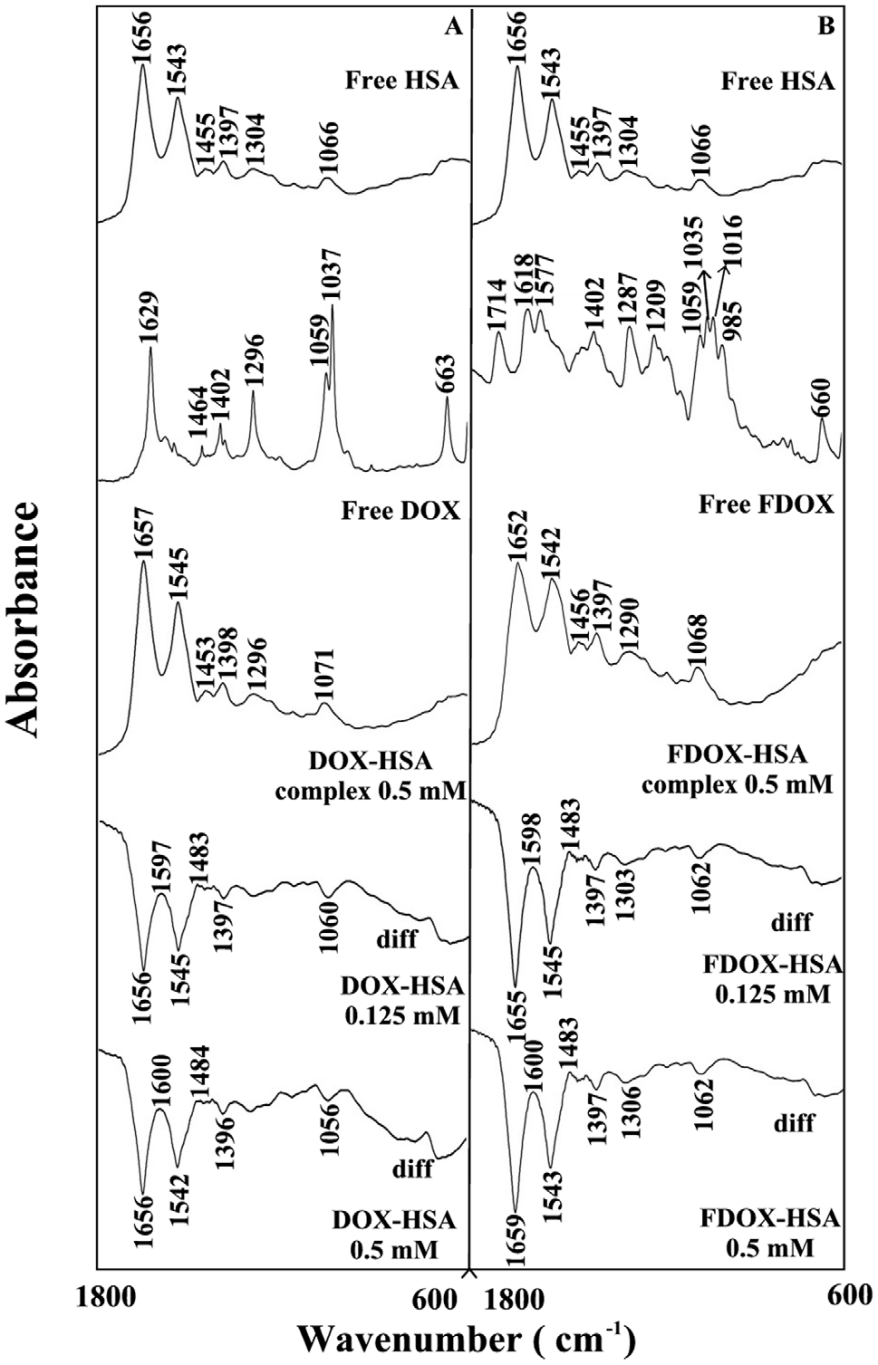
FTIR spectra in the region of 1800–600 cm^−1^ of hydrated films (pH 7.4) for free HSA (0.25 mM) and its drug complexes with difference spectra (diff.) (bottom two curves) obtained at different drug concentrations (indicated on the figure).

## Materials and Methods

### Materials

HSA and BSA fraction V were purchased from Sigma Chemical Company (St-Louis, MO) and used as supplied. Doxorubicin hydrochloride was generously provided by Pharmacia/Farmitalia Carlos Erba, Italy and *N*-(trifluoroacetyl) doxorubicin was synthesized according to the published methods [19], [20]. Other chemicals were of reagent grades and used as supplied.

**Figure 4:**
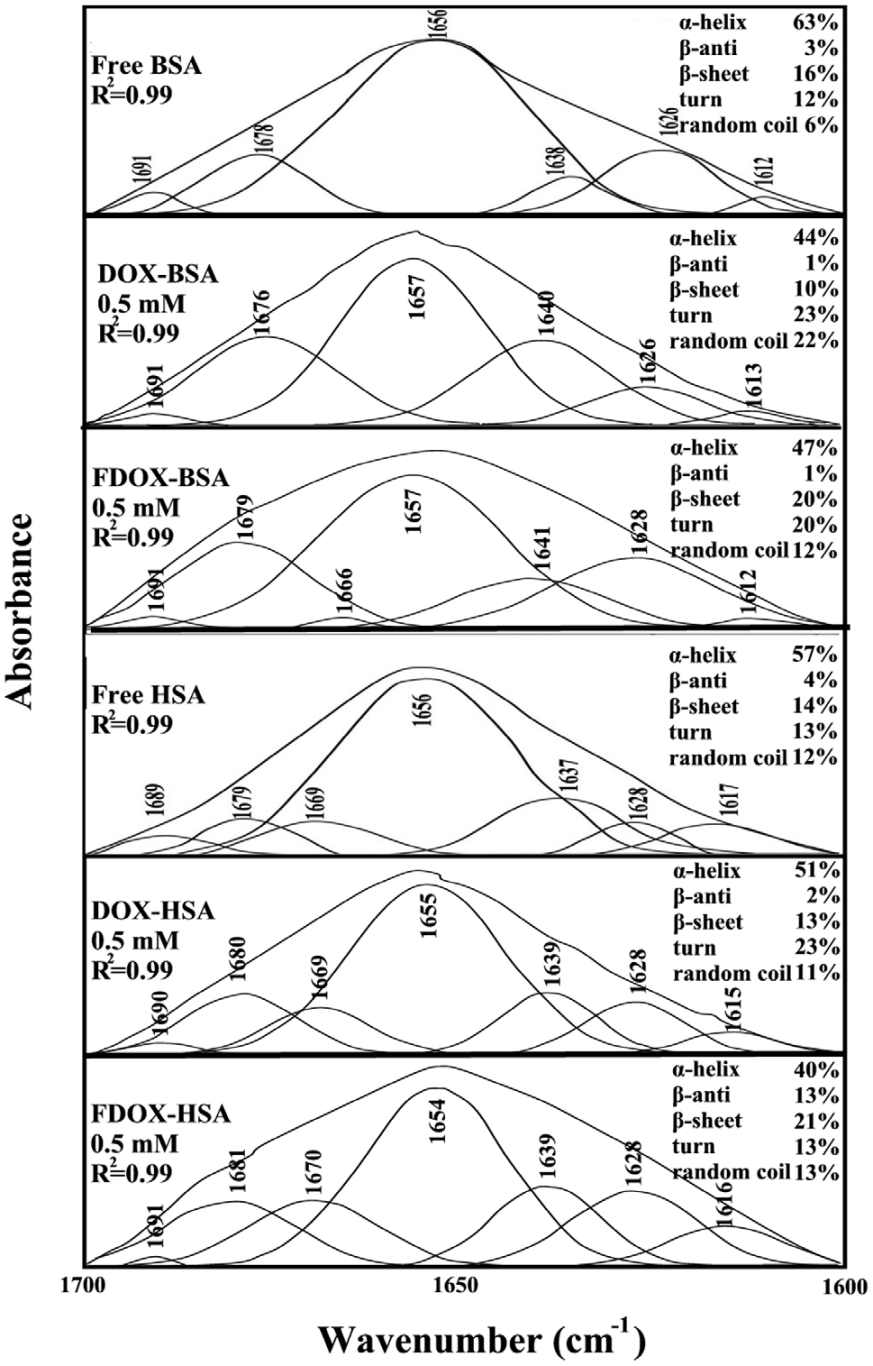
Second derivative resolution enhancement and curve-fitted amide I region (1700–1600 cm^−1^) for free BSA and HSA (0.25 mM) and their drug complexes with 0.5 mM drug concentration.

### Preparation of Stock Solutions

Protein (BSA or HSA) was dissolved in aqueous solution (40 mg/ml or 0.5 mM) containing 10 mM Tris-HCl buffers (pH 7.4). The protein concentration was determined spectrophotometrically using the extinction coefficient of 36 500 M^−1^ cm^−1^ at 280 nm [21]. A drug solution of 1 mM was prepared in 10 mM Tris-HCl and diluted to various concentrations in Tris-HCl (pH 7.4).

**Table 1:** Secondary structure analysis (infrared spectra) for the free BSA and HSA and their drug complexes in hydrated film at pH 7.4.

Amide I (cm^−1^)components	free BSA (%)0.25 mM	DOX (%)0.5 mM	FDOX(%)0.5 mM	free HSA (%)0.25 mM	DOX (%)0.5 mM	FDOX(%)0.5 mM)
α-helix (±4) 1654–1660	63	44	47	57	51	40
*β*-sheet (±2) 1614–1637	16	10	20	14	13	21
Random (±1) 1638–1648	6	22	12	12	11	13
turn (±2) 1670–1678	12	23	20	13	23	13
*β*-antiparallel (±1) 1680–1691	3	1	1	4	2	13

### FTIR Spectroscopic Measurements

Infrared spectra were recorded on a FTIR spectrometer (Impact 420 model), equipped with deuterated triglycine sulphate (DTGS) detector and KBr beam splitter, using AgBr windows. Solution of drug was added dropwise to the protein solution with constant stirring to ensure the formation of homogeneous solution and to reach the target drug concentrations of 0.125, 0.25 and 0.5 mM with a final protein concentration of 0.25 mM. Spectra were collected after 2 h incubation of BSA or HSA with drug solution at room temperature, using hydrated films. Interferograms were accumulated over the spectral range 4000–600 cm^−1^ with a nominal resolution of 1 cm^−1^ and 150 scans. The difference spectra [(protein solution + drug solution) – (protein solution)] were generated using water combination mode around 2300 cm^−1^, as standard [22]. When producing difference spectra, this band was adjusted to the baseline level, in order to normalize difference spectra.

**Table 2:** Secondary structure of BSA and HSA and their drug complexes (pH 7.4) calculated by CDSSTR Software (CD spectra).

Componentsconformation	free BSA (%)12.5 µM	DOX(%)0.5 mM	FDOX(%)0.5 mM	free HSA (%)12.5 µM	DOX(%)0.5 mM	FDOX(%)0.5 mM
*α*-helix (±3)	59	50	53	54	50	45
*β*-sheet (±2)	14	16	14	16	17	19
turn (±1)	10	15	15	15	16	16
random (±2)	16	19	18	15	17	20

### Analysis of Protein Conformation

Analysis of the secondary structure of BSA and HSA and their drug complexes was carried out on the basis of the procedure previously reported [23]. The protein secondary structure is determined from the shape of the amide I band, located around 1650–1660 cm^−1^. The FTIR spectra were smoothed and their baselines were corrected automatically using Grams AI software. Thus the root-mean square (rms) noise of every spectrum was calculated. By means of the second derivative in the spectral region 1700–1600 cm^−1^ six major peaks for BSA, HSA and complexes were resolved. The above spectral region was deconvoluted by the curve-fitting method with the Levenberg-Marquadt algorithm and the peaks corresponding to *α*-helix (1660–1654 cm^−1^), *β*-sheet (1637–1614 cm^−1^), turn (1678–1670 cm^−1^), random coil (1648–1638 cm^−1^) and *β*-antiparallel (1691–1680 cm^−1^) were adjusted and the areas were measured with the Gaussian function. The areas of all the component bands assigned to a given conformation were then summed up and divided by the total area [24], [25]. The curve-fitting analysis was performed using the GRAMS/AI Version 7.01 software of the Galactic Industries Corporation.

**Figure 5:**
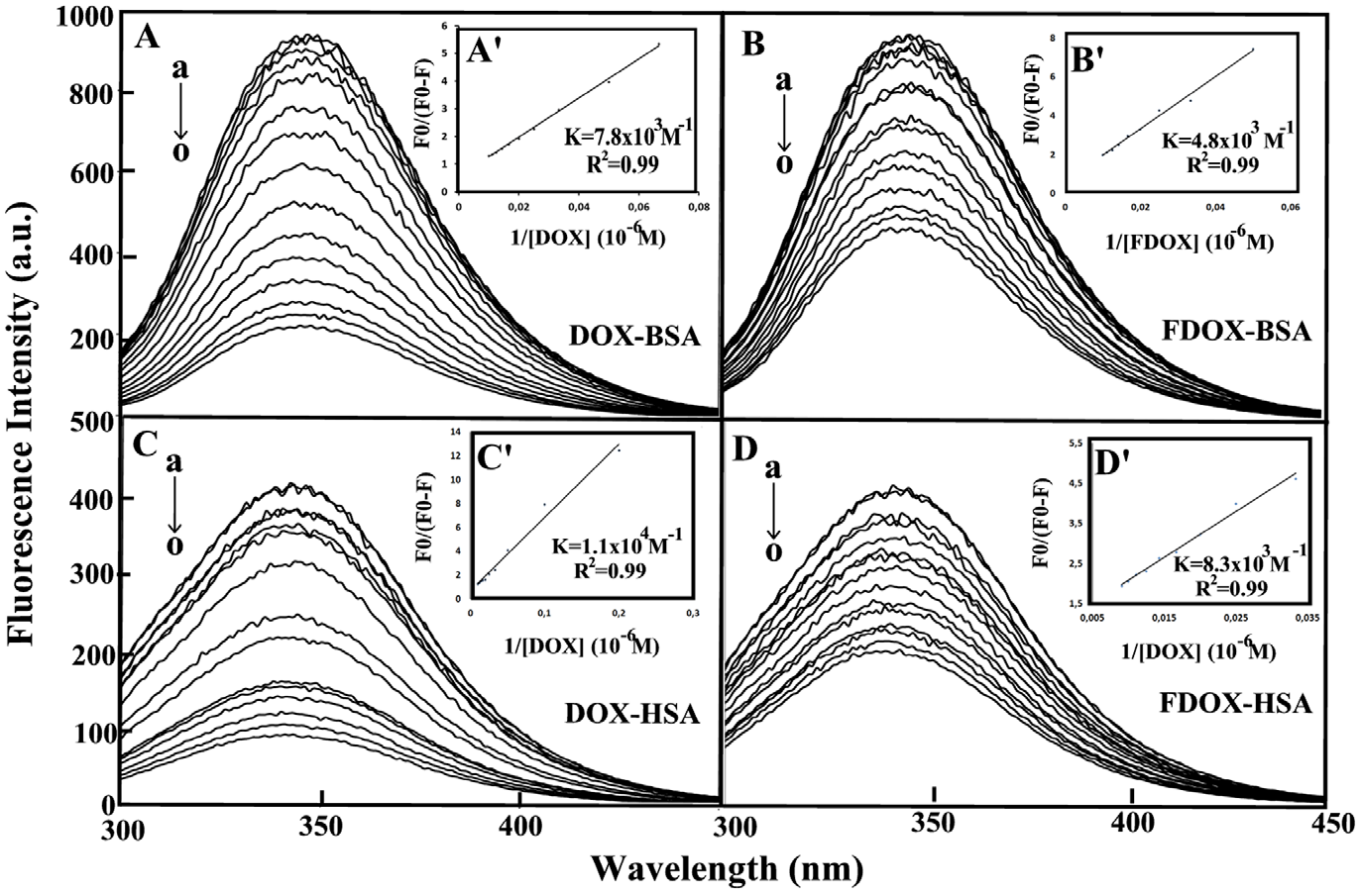
Fluorescence emission spectra of drug-BSA systems in 10 mM Tris-HCl buffer pH 7.4 at 25°C presented for (A) drug–BSA: (a) free BSA (10 µM), (b–o) with drug 1, 5, 7.5, 10, 15, 20, 30, 40, 50, 60, 70, 80, 90 and 100 µM; (B) drug– HSA: (a) free HSA (10 µM), (b–o) drug at 1, 5, 7.5, 10, 15, 20, 30, 40, 50, 60, 70, 80, 90 and 100 µM. Inset: *F*_0/_(*F*_0_–*F*) vs 1/[drug] for A’ (DOX-BSA), B’ (FDOX-BSA), C’ (DOX-HSA) and D’ (FDOX-HSA).

### Circular Dichroism

CD Spectra of BSA, HSA and their drug complexes were recorded with a Jasco J-720 spectropolarimeter. For measurements in the far-UV region (178–260 nm), a quartz cell with a path length of 0.01 cm was used in nitrogen atmosphere. Protein concentration was kept constant (12.5 µM), while varying drug concentrations (0.125, 0.25 and 0.5 mM). An accumulation of three scans with a scan speed of 50 nm per minute was performed and data were collected for each nm from 260 to 180 nm. Sample temperature was maintained at 25°C using a Neslab RTE-111 circulating water bath connected to the water-jacketed quartz cuvettes. Spectra were corrected for buffer signal and conversion to the Mol CD (Δε) was performed with the Jasco Standard Analysis software. The protein secondary structure was calculated using CDSSTR, which calculates the different assignments of secondary structures by comparison with CD spectra, measured from different proteins for which high quality X-ray diffraction data are available [26], [27]. The program CDSSTR is provided in CDPro software package which is available at the website: http://lamar.colostate.edu/~sreeram/CDPro.

**Figure 6:**
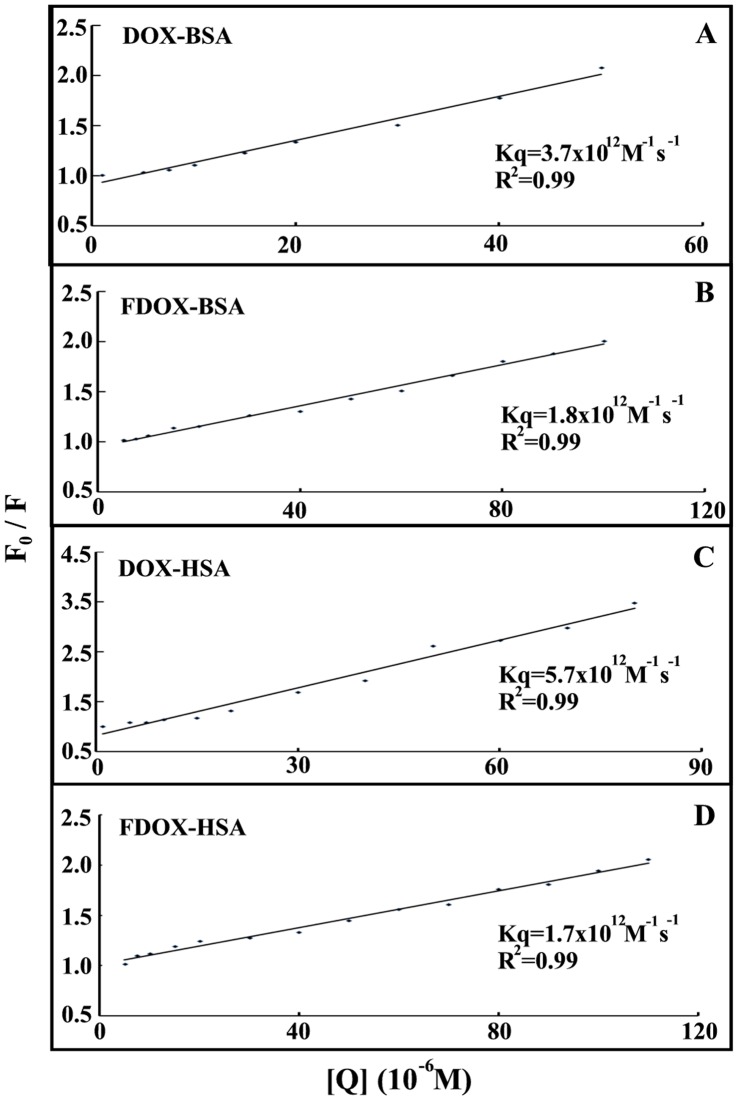
Stern-Volmer plots of fluorescence quenching constant (*K*q) for the drug-BSA and drug-HSA complexes at different drug concentrations (A) DOX-BSA and (B) FDOX-BSA (C) DOX-HSA and (D) FDOX-HSA.

### Fluorescence Spectroscopy

Fluorimetric experiments were carried out on a Varian Cary Eclipse. Solutions containing drug 1 to 100 µM in Tris-HCl (pH = 7.4) were prepared at room temperature (24±1°C). Solutions of HSA and BSA containing 10 µM in 10 mM Tris-HCl (pH = 7.4) were also prepared at 24±1°C. The fluorescence spectra were recorded at λ_exc_ = 280 nm and λ_em_ from 287 to 500 nm. The intensity at 347 nm (tryptophan) was used to calculate the binding constant (*K*) according to previous literature reports [28]–[33].

**Figure 7:**
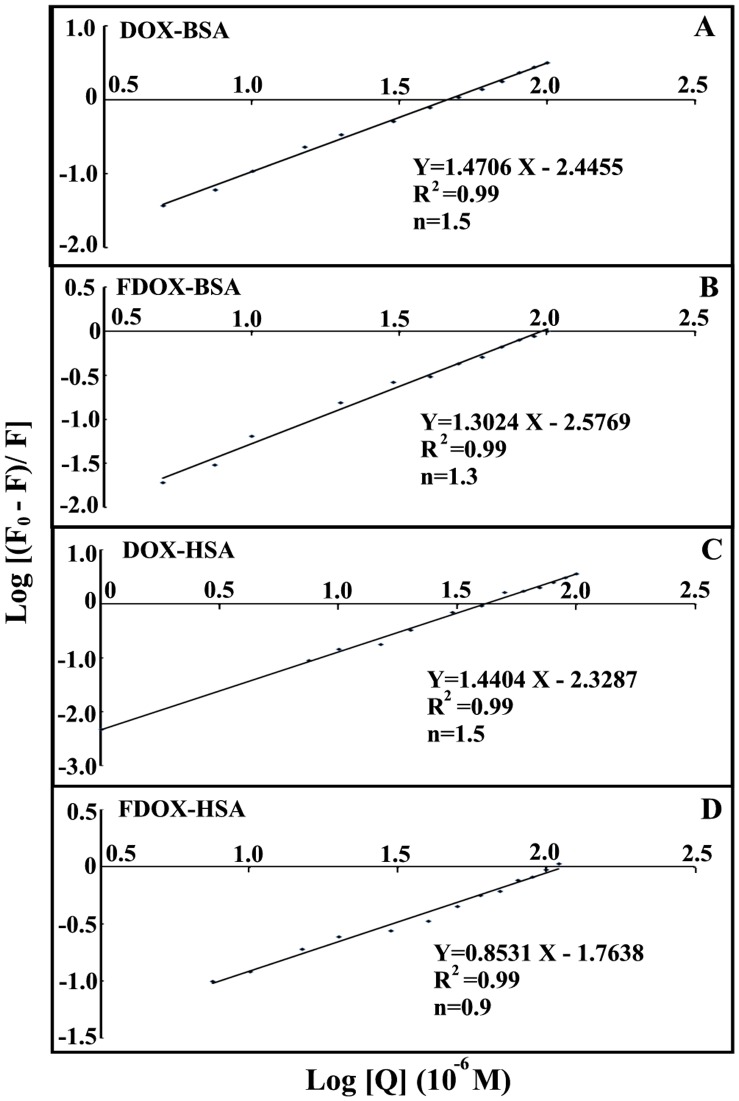
The plot of Log (*F_0_–F/F*) as a function of Log (drug concentration) and the number of bound drug molecules per protein (n).

On the assumption that there are (*n*) substantive binding sites for quencher (*Q*) on protein (*B*), the quenching reaction can be shown as following.
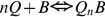
(1)


The binding constant (*K_A_*), can be calculated as:
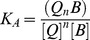
(2)Where [*Q*] and [*B*] are the quencher and protein concentration, respectively, [*Q_n_B*] is the concentration of non fluorescent fluorophore–quencher complex and [B_0_] gives total protein concentration.


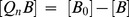
(3)


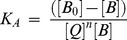
(4)

The fluorescence intensity is proportional to the protein concentration as follows:
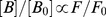
(5)


Results from fluorescence measurements can be used to estimate the binding constant of drug-protein complex. From eq 4:
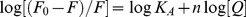
(6)


The accessible fluorophore fraction (*f*) can be calculated by modified Stern-Volmer equation.
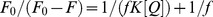
(7)Where *F*_0_ is initial fluorescence intensity and *F* is fluorescence intensities in the presence of quenching agent (or interacting molecule). *K* is the Stern-Volmer quenching constant, [Q] is the molar concentration of quencher and *f* is the fraction of accessible fluorophore to a polar quencher, which indicates the fractional fluorescence contribution of the total emission for an interaction with a hydrophobic quencher [17], [18]. The plot of *F_0_*_/_(*F_0_*–F) vs 1/[Q] yields *f* ^−1^ as the intercept on *y* axis and (*f K*)^−1^ as the slope. Thus, the ratio of the ordinate and the slope gives K.

**Figure 8:**
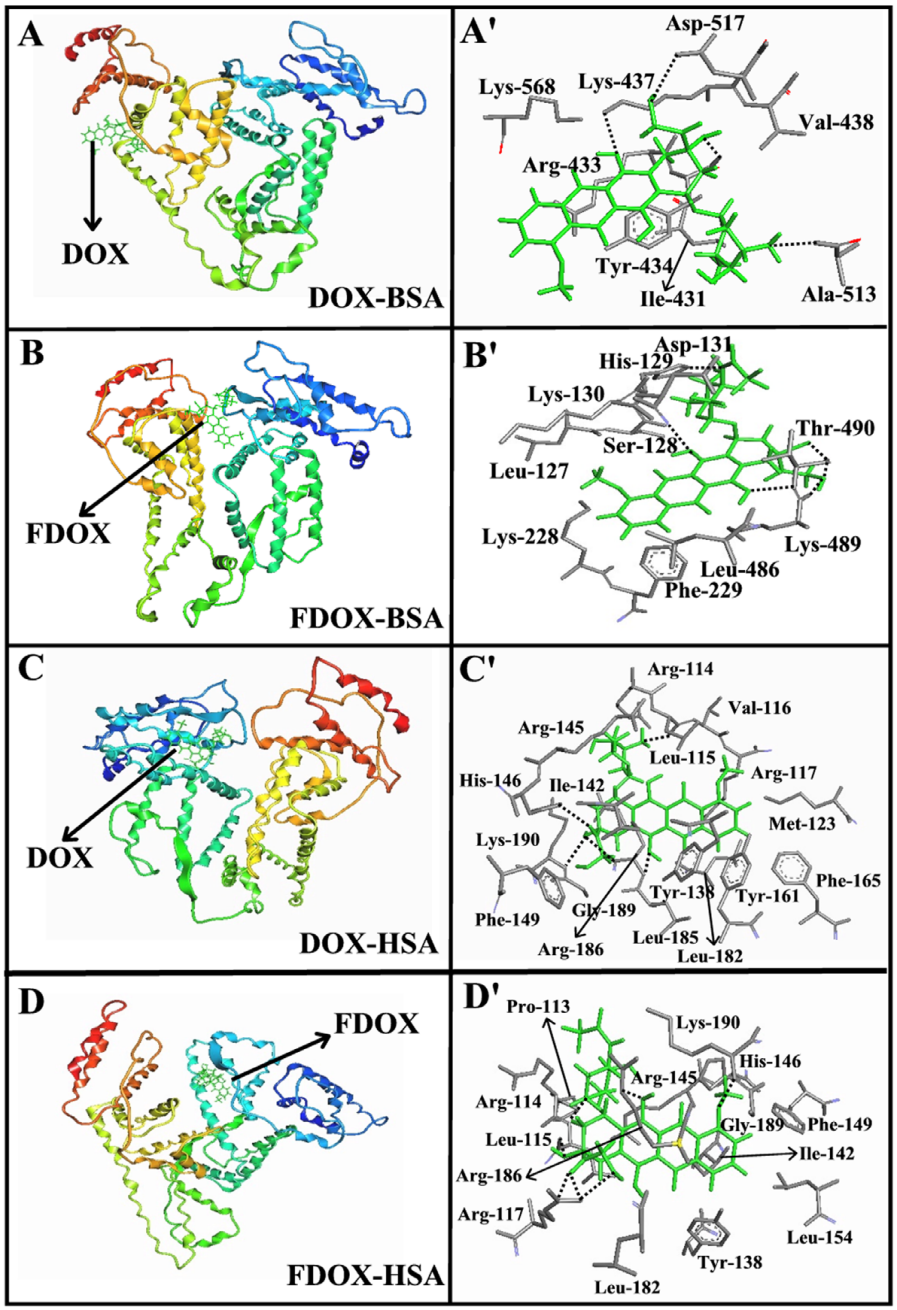
Best docked conformations of drug–BSA and drug–HSA complexes. (**A, A’**) for DOX complexed to BSA, (**B, B’**) for FDOX complexed to BSA, (**C, C’**) for DOX complexed to HSA, (**D, D’**) for FDOX complexed to HSA.

### Molecular Modeling

The structure of BSA was predicted by automated homology modelling using SWISS-MODEL Workspace [34] from the amino acid sequence NP-851335 [35], [36]. The structure of free HSA (PDB id: 1AO6, chain A) obtained by X-ray crystallography was used as a template [36]. These two proteins share 78.1% of sequence identity, which is sufficient to obtain reliable sequence alignment [37]. Images of the structures were generated using Pymol (DeLano Scientific, Palo Alto, CA, USA). RMSD between model and template proteins was 0.20 Å for positions of backbone atoms, as calculated with DeepView/Swiss-PdbViewer 4.0.1 (Fig. 1). The quality of the predicted BSA structure was found to be similar to the structure of free HSA used here as a template, using structure and model assessment tools of SWISS-MODEL workspace.

**Table 3:** Amino acid residues involved in drug-BSA and drug-HSA complexes with the free binding energy for the best selected docking positions.

Complex	Amino acids in the vicinity of drug	ΔG _binding_(Kcal/mol)
DOX-BSA	*Ala-513, Arg-433, *Asp-517, Ile-431,*Lys-437, Lys-568, *Tyr-434, Val-438.	−9.31
FDOX-BSA	Asp-131, *His-129, Leu-127, Leu-486,Lys-130, Lys-228, *Lys-489, Phe-229,*Ser-128, *Thr-490.	−9.06
DOX-HSA	Arg-114, Arg-117, Arg-145, *Arg-186,Gly-189, *His-146, Ile-142, *Leu-115,Leu-182, Leu-185, *Lys-190, Met-123,Phe-149, Phe-165, Tyr-138, Tyr-161,Val-116.	−10.75
FDOX-HSA	Arg-114, *Arg-117, Arg-145, *Arg-186,Gly-189, His-146, Ile-142, *Leu-115,Leu-154, Leu-182, *Lys-190, Phe-149,Pro-113, Tyr-138.	−10.37

* Hydrogen bonding involved with these amino acids.

The docking studies were performed with ArgusLab 4.0.1 software (Mark A. Thompson, Planaria Software LLC, Seattle, Wa, http://www.arguslab.com). The structure of BSA was obtained from the above method and three dimensional structure of drug was generated from PM3 semi-empirical calculations, using Chem3D Ultra 6.0. A blind docking approach was taken as the whole protein was selected as a potential binding site. The docking runs were performed on the ArgusDock docking engine using high precision with a maximum of 200 candidate poses. The conformations were ranked using the Ascore scoring function, which estimates the free binding energy. Upon location of the potential binding sites, the docked complex conformations were optimized using a steepest decent algorithm until convergence, within 40 iterations. Amino acid residues within a distance of 3.5 Å relative to drug were considered involved in the complexation.

## Results and Discussion

### FTIR Spectra of Drug Complexes with BSA and HSA

The drug interactions with BSA and HSA were characterized by infrared spectroscopy and its derivative methods. The spectral shifting and intensity variations of protein amide I band at 1656–1655 cm^−1^ (mainly C = O stretch) and amide II band at 1547–1543 cm^−1^ (C–N stretching coupled with N-H bending modes) [24], [33], [38] were monitored upon drug interaction. The difference spectra [(protein solution + drug solution) – (protein solution)] were obtained, in order to monitor the intensity variations of these vibrations and the results are shown in Figures 2 and 3. Similarly, the infrared self-deconvolution with second derivative resolution enhancement and curve-fitting procedures [23] were used to determine the protein secondary structures in the presence of drug (Figure 4 and Table 1).

At low drug concentration (0.125 mM), decrease of intensity was observed for the protein amide I at 1658–1656 and amide II at 1544–1543 cm^−1^, in the difference spectra of the drug-BSA and drug-HSA complexes (Figs. 2 and 3 A–B, diff. 0.125 mM). The negative features located in the difference spectra for amide I and II bands at 1659, 1546 cm^−1^ (DOX-BSA), 1661, 1549 cm^−1^ (FDOX-BSA) and 1656, 1545 cm^−1^ (DOX-HSA) and 1655, 1545 cm^−1^ (FDOX-HSA) are due to the loss of intensity of amide I and amide II bands upon drug interaction (Figs. 2 and 3 A–B, diff., 0.125 mM). This reduction of the intensity for the amide I and amide II bands is due to drug binding to protein C = O, C–N and N-H groups (hydrophilic contacts). Additional evidence to support the drug interactions with C–N and N-H groups comes from the shifting of the protein amide A band at 3290 cm^−1^ (N-H stretching) in the free HSA and BSA to higher frequency at 3310–3315 cm^−1^ upon lead drug interaction (spectra not shown). As drug concentration increased to 0.5 mM, strong negative features were observed for amide I band at 1661, 1549 (DOX-BSA), at 1660, 1550 (FDOX-BSA) and 1556, 1542 (DOX-HSA) and 1659, 1543 cm^−1^ (FDOX-HSA) upon drug complexation (Figs. 2 and 3 A–B, diff, 0.5 mM). In addition, spectral shifting was observed for the amide I at 1658–1656 and amide II at 1544–1543 cm^−1^ upon drug-protein complexation (Figs. 2 and 3, A–B, 0.5 mM complexes). The observed spectra shifting amide I and amide II bands are due to drug binding to protein C–O and C–N groups, while the decrease in the intensity of the amide I band in the spectra of the drug-protein complexes suggests a major reduction of protein α-helical structure at high drug concentrations [39].

A quantitative analysis of the protein secondary structure for the free BSA, HSA and their drug complexes in hydrated films has been carried out and the results are shown in Figure 4 and Table 1. The free BSA contains α-helix 63% (1656 cm^−1^), *ß-*sheet 16% (1612 and 1626 cm^−1^), turn 12% (1678 cm^−1^), *ß*-antiparallel 3% (1691 cm^−1^) and random coil 6% (1638 cm^−1^) (Fig. 4A and Table 1) consistent with the conformation of BSA reported [40], [41]. The free HSA has 57% *α*-helix (1656 cm^−1^), *ß*-sheet 14% (1628 and 1617 cm^−1^), turn structure 13% (1669 cm^−1^), *ß*-antiparallel 4% (1689 cm^−1^) and random coil 12% (1637 cm^−1^) (Fig. 4C and Table 1) consistent with the spectroscopic studies of human serum albumin [24], [29]. Upon drug interaction, a major decrease of *α*-helix from 63% (free BSA) to 47–44% (drug-complex) with increase in random coil and turn structure were observed (Fig. 4B and Table 1). Similarly, a major decrease of *α*-helix from 57% (free HSA) to 51–40% (drug complex) also occurred on drug-HSA complexation (Fig. 4 C and D). It should be noted that the spectral changes were more pronounced for drug-BSA than drug-HSA complexes.

### CD Spectra

The conformational changes observed from infrared results for BSA and HSA and their drug complexes are consistent with CD spectroscopic analysis shown in Table 2. The CD results show that free BSA has a high α-helix content 59%, β-sheet 14%, turn 11% and random coil 16% (Table 2), consistent with the literature report [41]. The free HSA contains α-helix 54%, β-sheet 16%, turn 15% and random coil 15% (Table 2). Upon drug complexation, major reduction of α-helix was observed from 59% in free BSA to 53–50% in drug-BSA and from 54% to 50–45% in the drug-HSA complexes (Table 2). The decrease in α-helix was accompanied by an increase in the β-sheet, turn and random coil structures (Table 2). The major reduction of the α-helix with an increase in the β-sheet, turn and random structures are consistent with the infrared results, indicating a partial protein destabilization (Tables 1 and 2). However, the drugs induced more perturbations of HSA structure than that of BSA conformation (Table 1) consistent with more stable drug-HSA complexes than those of drug-BSA adducts, which will be discussed in fluorescence spectroscopy.

### Fluorescence Spectra and Stability of Drug Complexes with BSA and HSA

HSA contains a single polypeptide of 585 amino acids with only one tryptophan (Trp-214) located in subdomain II A. BSA contains two tryptophan residues Trp-134 and Trp-212 located in the first and second domains of protein hydrophobic regions. Tryptophan emission dominates both HSA and BSA fluorescence spectra in the UV region. The decrease of fluorescence intensity of HSA and BSA has been monitored at 347 nm for drug-protein systems (Fig. 5A–D show representative results for each system). The plot of *F*_0/_(*F*_0_–*F*) vs 1/[drug] (Fig. 5A’–D’ show representative plots for drug-protein complexes). Assuming that the observed changes in fluorescence come from the interaction between drug and HSA or BSA, the quenching constant can be taken as the binding constant of the complex formation. The *K* values given here are averages of four-replicate and six-replicate runs for drug-protein systems, each run involving several different drug concentrations (Fig. 5A–D). The binding constants obtained were *K*_DOX-BSA_ = 7.8 (±0.7)×10^3^ M^−1^, *K*_FDOX-BSA_ = 4.8 (±0.5)×10^3^ M^−1^ and *K*_DOX-HSA_ = 1.1 (±0.3)×10^4^ M^−1^, *K*_FDOX-HSA_ = 8.3 (±0.6)×10^3^ M^−1^ (Fig. 5A’–D’). The association constants calculated for the drug complexes suggest strong affinity for drug-protein binding, compared to the other ligand-protein adducts [42], [43]. However, HSA forms more stable complexes than BSA due to major hydrophilic and hydrophobic drug-HSA interactions.

In order to verify the presence of static or dynamic quenching in drug-protein complexes we have plotted *F_0_/F* against *Q* and the results are show in Fig. 6. The plot of *F_0_/F* versus Q is linear for drug-BSA and drug-HSA adducts indicating that the quenching is mainly static in these drug-protein complexes [44]. The *K*_q_ was estimated according to the Stern-Volmer equation:
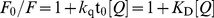
(8)where *F_0_* and *F* are the fluorescence intensities in the absence and presence of quencher, [Q] is the quencher concentration and *K*_D_ is the Stern-Volmer quenching constant (*K_q_*), which can be written as *K*_D_ = k_q_t_0_; where *k_q_* is the bimolecular quenching rate constant and t_0_ is the lifetime of the fluorophore in the absence of quencher, 5.9 ns for BSA and 5.6 ns for HSA [17]. The quenching constants (*K_q_*) are 3.7×10^12 ^M^−1^/s for DOX-BSA and 1.7×10^12 ^M^−1^/s for FDOX-BSA, 5.7×10^12 ^M^−1^/s for DOX-HSA and 1.6×10^12 ^M^−1^/s for FDOX-HSA complexes (Fig. 6A–D). Since these values are much greater than the maximum collisional quenching constant (2.0×10^10 ^M^−1^/s), thus the static quenching is dominant in these drug-protein complexes [44].

The number of drug molecule bound per protein (*n*) is calculated from log [(*F*_0_–*F*)/*F*] = log *K*_S_ + *n* log [drug] for the static quenching [45]–[49]. The linear plot of log [(*F*_0_–*F*]/*F*] as a function of log [drug] is shown in Figure 7A–D. The *n* values from the slope of the straight line are 1.5 (DOX-BSA) and 1.3 (F-DOX-BSA) 1.5 (DOX-HSA), 0.9 (FDOX-HSA), in these drug-protein complexes (Fig. 7A–D).

### Docking Studies

Our spectroscopic results were complemented with docking experiments in which doxorubicin and *N*-(trifluoroacetyl) doxorubicin were docked to BSA and HSA to determine the preferred binding sites for DOX and FDOX. The docking results shown in Fig. 8 and Table 3 are related to both drug-BSA and drug-HSA complexes. In the DOX-BSA complexes, drug is surrounded by *Ala-513 (2.99 Å = H-bond), Arg-433, *Asp-517 (2.85 Å = H-bond), Ile-431, *Lys-437 (2.53 Å = H-bond), Lys-568, *Tyr-434 (2.34 Å = H-bond) and Val-438 with the binding energy of −9.31 kcal/mol (Fig. 8 and Table 3). In the FDOX-BSA, drug is located near Asp-131, *His-129 (2.99 Å = H-bond), Leu-127, Leu-486, Lys-130, Lys-228, *Lys-489 (2.80 Å = H-bond), Phe-229, *Ser-128 (2.42 Å = H-bond) and Thr-490 with the binding energy of −9.06 kcal/mol (Fig. 8 and Table 3). On the other hand, in DOX-HSA complexes, DOX is surrounded by Arg-114, Arg-117, Arg-145, *Arg-186 (2.72 Å = H-bond), Gly-189, *His-146 (2.66 Å = H-bond), Ile-142, *Leu-115 (2.11 Å = H-bond), Leu-182, Leu-185, *Lys-190 (2.86 Å = H-bond), met-123, Phe-149, Phe-165, Tyr-138, Tyr-161-and Val-116 with the free binding energy of −10.75 kcal/mol (Fig. 8 and Table 3). Similarly, in FDOX-HSA, drug is located in the vicinity of Arg-114, *Arg-117 (2.92 Å = H-bond), Arg-145, *Arg-186 (2.84 Å = H-bond), Gly-189, His-146, Ile-142, *Leu-115 (2.90 and 296 Å = H-bonds), Leu-154, leu-182, *Lys-190 (2.73 Å = H-bond), Phe-149, Pro-113 and Tyr-139 with the free binding energy of −10.37 kcal/mol (Fig. 8 and Table 3). It is evident that several amino acids with hydrophobic and hydrophilic characters are in contact with DOX and FDOX in these BSA and HSA complexes (Fig. 8 and Table 3). Molecular modeling presented in Table 3 showed major differences in the binding sites of DOX and FDOX with BSA and HSA (different amino acids are involved in drug-protein complexation). Docking results showed stronger drug-protein complex formed with HSA than BSA which is consistent with our spectroscopic results *K*_DOX-HSA_ = 1.1×10^4^ M^−1^, *K*_FDOX-HSA_ = 8.3×10^3^ M^-1^, *K*_DOX-BSA_ = 7.8×10^3^ M^−1^ and *K*_FDOX-BSA_ = 4.8×10^3^ M^−1^. (Fig. 5A’–D’).

### Conclusion

Based on our spectroscopic and docking results, doxorubicin and *N*-(trifluoroacetyl) doxorubicin bind BSA and HSA *via* hydrophilic and hydrophobic contacts with more stable complexes formed with HSA than BSA. The drug-protein binding involves several amino acids residues, which stabilized by H-bonding network. Drug interaction alters protein secondary structure for both BSA and HSA causing a partial protein destabilization. It is clearly shown that HSA and BSA can transport DOX and FDOX *in vitro*.
